# Climate Variability, Weather and Enteric Disease Incidence in New Zealand: Time Series Analysis

**DOI:** 10.1371/journal.pone.0083484

**Published:** 2013-12-23

**Authors:** Aparna Lal, Takayoshi Ikeda, Nigel French, Michael G. Baker, Simon Hales

**Affiliations:** 1 Department of Public Health, University of Otago, Wellington, New Zealand; 2 Dean’s Department, University of Otago, Wellington, New Zealand; 3 Molecular Epidemiology and Public Health laboratory, Hopkirk Research Institute, Massey University, Palmerston North, New Zealand; University of Oxford, Viet Nam

## Abstract

**Background:**

Evaluating the influence of climate variability on enteric disease incidence may improve our ability to predict how climate change may affect these diseases.

**Objectives:**

To examine the associations between regional climate variability and enteric disease incidence in New Zealand.

**Methods:**

Associations between monthly climate and enteric diseases (campylobacteriosis, salmonellosis, cryptosporidiosis, giardiasis) were investigated using Seasonal Auto Regressive Integrated Moving Average (SARIMA) models.

**Results:**

No climatic factors were significantly associated with campylobacteriosis and giardiasis, with similar predictive power for univariate and multivariate models. Cryptosporidiosis was positively associated with average temperature of the previous month (β =  0.130, SE =  0.060, p <0.01) and inversely related to the Southern Oscillation Index (SOI) two months previously (β =  −0.008, SE =  0.004, p <0.05). By contrast, salmonellosis was positively associated with temperature (β  = 0.110, SE = 0.020, p<0.001) of the current month and SOI of the current (β  = 0.005, SE = 0.002, p<0.050) and previous month (β  = 0.005, SE = 0.002, p<0.05). Forecasting accuracy of the multivariate models for cryptosporidiosis and salmonellosis were significantly higher.

**Conclusions:**

Although spatial heterogeneity in the observed patterns could not be assessed, these results suggest that temporally lagged relationships between climate variables and national communicable disease incidence data can contribute to disease prediction models and early warning systems.

## Introduction

Global climate change is projected to increase the frequency and intensity of extreme climatic events such as floods, droughts and cyclones [1], [2]. These climatic conditions have been associated with increased enteric disease risk [3]–[7]. Moreover, atypical weather known to accompany climatic phenomena such as *El Niño* have been implicated in enteric disease outbreaks worldwide [8]. Despite this evident association with climatic factors, our understanding of the impacts of regional climate variability on infectious disease risk is driven primarily by research focussed on mosquito borne diseases such as malaria [9] and dengue [10] and diseases such as cholera [11] and influenza [12]. Examining the associations between regional climate linked to the El Niño/Southern Oscillation (ENSO) and enteric disease will develop our understanding of climatic triggers for enteric infections as well as improve disease forecasts.

The effect of ENSO on global climate, through inter-annual fluctuations in temperature, precipitation and atmospheric circulation at distant locations is termed teleconnection. The New Zealand climate shows a moderate teleconnection to ENSO at seasonal to interannual scales, with generally cooler and drier conditions during the *El Niño* phase and warmer and wetter conditions during the *La Niña* phase [13]. Such climatic variations are likely to affect enteric disease incidence either directly, through effects on pathogen competence, or indirectly, by influencing transmission pathways and host behaviour [14], [15].

While locality specific impacts of climate change on disease risk will depend on a number of interacting climatic and non-climatic factors; larger scale, regional disease patterns are more likely to be dominated by extrinsic climate forcing [16]. To date, there are no published studies taking a comparative approach to assessing the influence of such large scale environmental processes across multiple diseases over an entire region. Such an analysis would enable comparisons with other regions [17], [18]. This would allow the impacts of global climate variability and change on enteric disease risk to be better evaluated.

New Zealand reports some of the highest enteric disease rates among industrialised countries [19]. Associations between temperature and salmonellosis [20] and rainfall and cryptosporidiosis and giardiasis [21], [22] as well as distinct seasonal disease patterns [16] suggest that climate variability is an important determinant of enteric disease. Importantly, the need for increased research around climate change and infectious disease risks to inform adaptation responses in New Zealand has been identified as a priority [23].

Seasonal Auto-Regressive Moving Average (SARIMA) modelling is a statistical approach to model and forecast time series which are non-stationary and where the observations are seasonally dependant and autocorrelated [24]; key characteristics of our dataset. When modelling the association between climate variation and cryptosporidiosis incidence in Australia, model assessments indicated that the seasonal ARIMA model had better predictive ability than Poisson models [25], with similar results obtained when comparing regression models for salmonellosis transmission in Australia [26]. In this study, we use SARIMA models to analyse national enteric disease incidence in relation to ENSO as measured by the Southern Oscillation Index (SOI) and weather variability (average monthly temperature and rainfall).

## Methods

### Case data

All notified, laboratory confirmed cases of campylobacteriosis, salmonellosis, cryptosporidiosis and giardiasis during the period 1997–2008 in New Zealand were obtained from the National Notifiable Disease Surveillance system (EpiSurv), operated by the Institute of Environmental Science and Research (ESR) for the Ministry of Health. To better evaluate the temporal pattern of associations between climate and disease notifications, only cases with a reported onset date were used. In total, 79193 cases of campylobacteriosis, 14084 cases of (non-typhoidal) salmonellosis, 8092 cases of cryptosporidiosis and 10424 cases of giardiasis were included in the analysis.

Using onset date, cases were aggregated into counts by month over the study period. National incidence rates were calculated using the monthly number of cases as the numerator and the 2001 census population as the denominator [27]. To lessen the effect of extreme values (e.g. outbreaks) on model outcomes and to normalise the data, the natural logarithm of monthly incidence rates was used (hereafter referred to as monthly incidence).

### Climate data

For 1997–2008, daily surface average temperature (°C) and average rainfall (millimetres) were obtained from surface temperature and precipitation time series records constructed from gridded climate data and spatially averaged over New Zealand as specified by latitude (35.25S) to (47.75S) and longitude (166.25E) to (177.75E) [28]. This data source has been used extensively for the association of climatic factors with vector borne diseases in the Pacific [29] vector distribution in Gambia [30] and salmonellosis in New Zealand [20]. The daily values were aggregated monthly.

The Southern Oscillation Index (SOI) is the most commonly used index to measure the intensity of an ENSO event. It is based on the differences in atmospheric pressure between Tahiti in the eastern equatorial Pacific and Darwin, Australia in the west Pacific, expressed as a standard deviation from the norm. Negative anomalies are generally associated with El Niño events and positive anomalies with La Niña events. The monthly SOI was obtained from the Australian Bureau of Meteorology. All climate data covered the same time intervals as the disease data.

### Data analysis

In order to investigate delayed effects of climate variability on disease outcomes, climate variables were temporally lagged by up to two months. All climate variables that showed a cross correlation with disease incidence up to a lag of two months were included in the model. A multivariate seasonal autoregressive integrated moving average (SARIMA) model was used to examine the combined effect of climatic variables on enteric disease incidence for each of the diseases separately. As both the dependent and independent variables exhibited periodicity, they were seasonally differenced before analysis (as described below).

### Model specification

Using incidence data from 1997–2007, a SARIMA model was fitted to disease data and used to predict incidence rates for each of the four diseases in 2008 [31]. To check for seasonal effects, the time series plot of monthly incidence was examined and an Augmented Dickey-Fuller (ADF) test was used. To achieve a stationary time series, monthly incidence was seasonally differenced by replacing each observation by the difference between itself and the observation a year previously. The climatic variables were also seasonally differenced.

To examine the independent contribution of climatic variables to enteric disease incidence a Seasonal ARIMA model that includes seasonality, referred to as SARIMA(p, d, q)(P, D, Q), where p denotes the AR order, d the differencing order and q the MA order that was used. P, D and Q denote the seasonal order of AR, differencing, and MA, respectively. Akaike’s Information Criterion (AIC) was used to assist model selection [32]. To check for seasonal effects, and help identify the model parameters, the Autocorrelation function (ACF) and partial autocorrelation function (PACF) were analysed. The residuals were further examined for autocorrelation using ACF and PACF.

Goodness of fit was examined through Portmanteau test for white noise in residuals and a scatter plot of residuals versus fitted values. Furthermore, the disease dataset was divided into two: one (1997–2007) was used for the model fitting process (parameter estimation), and another for prediction (2008). To verify model fit, the predictive ability of both models (with and without climatic variables) was assessed using the Diebold- Mariano test, which tests the null hypothesis of equal accuracy using Mean Absolute Percentage Error (MAPE) and Mean Absolute Error (MAE). Lower MAPE and MAE values indicate a better fit of the data. Finally, a plot of time series on the cumulative sums of actual and predicted values was used to assess model validity. All of the analyses were conducted using STATA v11.1 (StataCorp LP, College Station, TX, USA).

## Results

### Descriptive analyses

Descriptive statistics for the disease notification and weather variables are presented in Table 1. Correlations between the differenced independent variables indicate that relationships between monthly surface temperature, rainfall and SOI were neither strong nor statistically significant (Table 2).

**Table 1:** Descriptive statistics for the disease and climatic variables in New Zealand, during 1997–2008.

Variable	Mean ±SD	Minimum	Maximum
Campylobacteriosis incidence*	14.90±5.88	4.84	34.33
Salmonellosis incidence*	2.85±1.24	0.96	6.74
Cryptosporidiosis incidence*	1.64±1.43	0.24	5.94
Giardiasis incidence*	1.93±0.48	0.96	3.48
Rainfall (mm)	142.16±35.50	63.00	240.39
Temperature (°C)	10.71±3.46	4.63	17.62
SOI	1.98±14.79	–37.70	42.90

* Average monthly incidence /100000 population.

**Table 2:** Spearman’s correlation coefficients between independent climatic variables.

Variable		Temperature (°C)	SOI
Rainfall (mm)		0.13	0.007
Temperature (°C)			0.01

### Model specification

**Figure 1:**
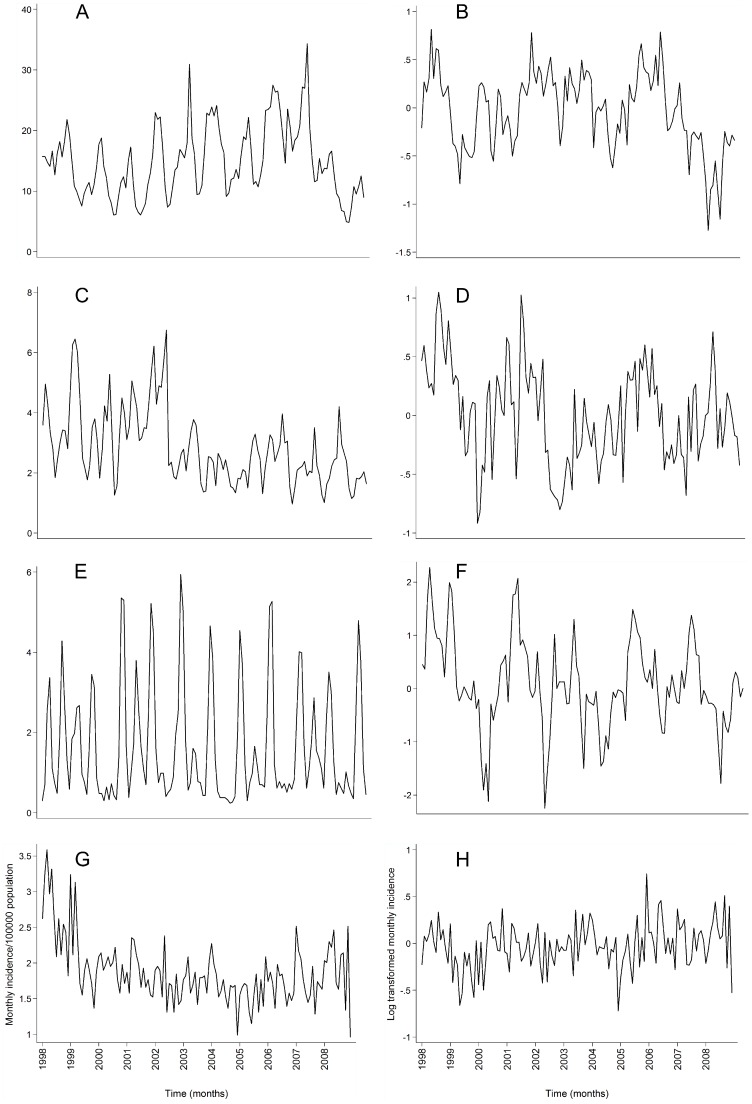
Time series of raw and log transformed monthly incidence (after differencing) of campylobacteriosis (A-B), salmonellosis (C-D), cryptosporidiosis (E-F), and giardiasis (G-H) in New Zealand, 1997-2008.

The log transformed and differenced time series showed less periodicity than the original monthly incidence, with no apparent trend (Figure 1). The transformed time series were considered stationary based on ADF tests (Table 3).

**Table 3:** Results of the Augmented Dickey-Fuller test of the transformed, seasonally differenced time series for all four diseases.

Variable†	Dickey-Fuller test for unit root			
	t_α_ ‡	1%	5%	10%
Campylobacteriosis	–3.97**	–3.50	–2.88	–2.57
Salmonellosis	–5.39**	–3.50	–2.88	–2.57
Cryptosporidiosis	–4.18**	–3.50	–2.88	–2.57
Giardiasis	–10.00**	–3.50	–2.88	–2.57

† Log-transformed and seasonally differenced monthly incidence /100000 population.

** Significant at the 0.01 level.

‡ is the computed ADF test statistic which is compared to the critical values at significant levels α = 0.01, 0.05 and 0.1. If the test statistic is less than the critical value, then the null hypothesis is rejected, and thus the variable is stationary.

For campylobacteriosis, the best model was SARIMA (1, 0, 0) (2, 0, 0)_12_ (Log-likelihood  =  23.63, AIC  = –37.26) (Table 4). Campylobacteriosis incidence was positively associated with the temperature of the previous two months, but there was no relationship with precipitation or SOI (Table 5). The model estimated without the climatic variables was a slightly better fit than the model with these variables (Table 4).

**Table 4:** Regression coefficients of the chosen SARIMA models (with and without climatic predictors) on the monthly incidences of campylobacteriosis, salmonellosis, cryptosporidiosis and giardiasis in New Zealand.

Variable	Model without climate variables ^abcd^			Model with climate variables ^efgh^		
	β	SE	p-value	β	SE	p-value
CAMPYLOBACTERIOSIS						
Autoregression	0.79	0.05	<0.001	0.79	0.06	<0.001
Seasonal autoregression (1)	–0.73	0.09	<0.001	–0.73	0.10	<0.001
Seasonal autoregression (2)	–0.28	0.10	<0.01	–0.28	0.10	<0.01
Temperature -2months previous				0.01	0.01	0.48
SALMONELLOSIS						
Autoregression	0.71	0.07	<0.001	0.63	0.07	<0.001
Seasonal autoregression	–0.50	0.06	<0.001	–0.48	0.07	<0.01
Temperature current month				0.11	0.02	<0.001
SOI current month				0.005	0.002	<0.05
SOI previous month				0.005	0.002	<0.05
CRYPTOSPORIDIOSIS						
Autoregression	0.75	0.04	<0.001	0.73	0.05	<0.001
Seasonal autoregression	–0.56	0.08	<0.001	–0.61	0.08	<0.001
Temperature previous month				0.13	0.04	<0.01
SOI -2months previous				–0.008	0.004	<0.05
GIARDIASIS						
Autoregression	0.44	0.08	<0.001	0.39	0.08	<0.001
Seasonal autoregression	–0.24	0.11	<0.05	–0.24	0.13	0.06
Seasonal moving average	–0.85	0.23	<0.001	–0.78	0.18	<0.001
Temperature current month				0.02	0.01	0.14
Precipitation current month				–0.0004	0.0003	0.29
SOI -2months previous				–0.001	0.001	0.40

a Campylobacteriosis, log-likelihood  =  23.63, AIC  = –37.26.

b Salmonellosis, log-likelihood  =  –20.37, AIC  = 48.74.

c Cryptosporidiosis, log-likelihood  =  –78.53 AIC  =  165.06.

d Giardiasis, log-likelihood  =  39.10 AIC  =  –68.20.

e Campylobacteriosis, log-likelihood  =  23.39, AIC  = –34.79.

f Salmonellosis, log-likelihood  =  –4.65, AIC  =  29.31.

g Cryptosporidiosis, log-likelihood  =  –68.00, AIC  =  154.00.

h Giardiasis, log-likelihood  =  39.48 AIC  =  –62.97.

**Table 5:** Spearman’s rank cross correlation coefficients of (seasonally differenced) disease incidence and climatic variables in New Zealand.

Variable	Lag0	Lag1	Lag2
CAMPYLOBACTERIOSIS			
Temperature	-	-	0.15
Rainfall	-	-	-
SOI	-	-	-
SALMONELLOSIS			
Temperature	0.46	0.21	0.32
Rainfall	-	-	-
SOI	0.31	0.30	0.32
CRYPTOSPORIDIOSIS			
Temperature	0.27	0.20	0.16
Rainfall	-0.15	-	-
SOI	-	-	0.24
GIARDIASIS			
Temperature	0.13	-	-
Rainfall	-0.12	-	-
SOI	-	-	0.24

For salmonellosis, a SARIMA (1, 0, 0) (1, 0, 0)_12_ model was the best fit (Log-likelihood  =  –20.37, AIC  = 48.74) (Table 4). The temperature and SOI of the current month, and lagged by 1 and 2 months were associated with incidence, but there was no relationship with precipitation (Table 5). The model estimated with the climatic variables was a better fit than the model without these variables (i.e. the log-likelihood increased, while AIC decreased) (Table 4).

For cryptosporidiosis, the best fitting model was SARIMA (1, 0, 0) (1, 0, 0)_12_ (Log-likelihood  =  –78.53, AIC  = 165.06) (Table 4). Temperature and precipitation of the current month, temperature (lagged by 1 and 2 months) and the SOI (lagged by 2 months) were each associated with cryptosporidiosis onset (Table 5). The model estimated with the climatic variables was a better fit than the model without these variables (Table 4).

For giardiasis, the best model was SARIMA (1, 0, 0) (1, 0, 1)_12_ (Log-likelihood  =  –20.37, AIC  = 48.74) (Table 4). The temperature and precipitation of the current month and SOI of the previous 2 months were associated with disease (Table 5). There was no apparent difference in models estimated with the climatic variables compared to models without these variables (Table 4).

**Figure 2:**
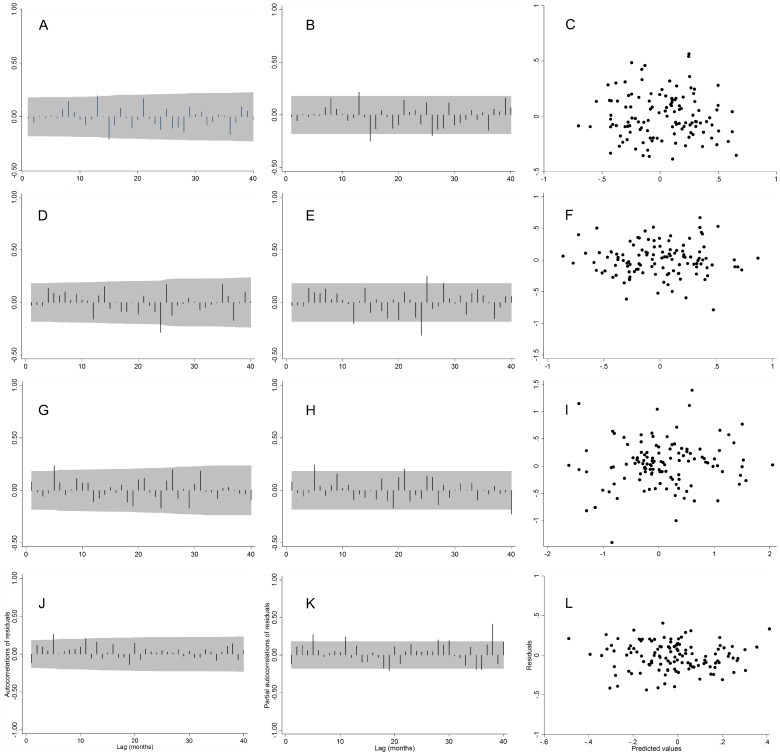
Autocorrelation plots, partial autocorrelation plots of the residuals and scatter plot of residuals against the predicted values of the seasonal autoregressive moving average SARIMA model fitted to the natural logarithm differenced disease incidence. Campylobacteriosis SARIMA (1, 0, 0) (2, 0, 0)_12_ (A-C), salmonellosis SARIMA (1, 0, 0) (1, 0, 0)_12_ (D-F), cryptosporidiosis SARIMA (1, 0, 0) (1, 0, 0)_12_ (G-I), giardiasis SARIMA (1, 0, 0) (1, 0, 1)_12_ (J-L). The x-axis gives the number of lags in months and the grey shaded areas represent the 95% confidence interval.

For all four diseases, the plots of the ACF and PACF of the residuals of the chosen models showed no significant temporal correlation between residuals at different lags (Figure 2) and the scatter plot of the predicted values against the residuals showed no discernible pattern (Figure 2). Portmanteau Q statistics for campylobacteriosis, salmonellosis, cryptosporidiosis, and giardiasis of 51.22, 53.09, 51.22 and 41.85, respectively.

### Validation model

**Figure 3:**
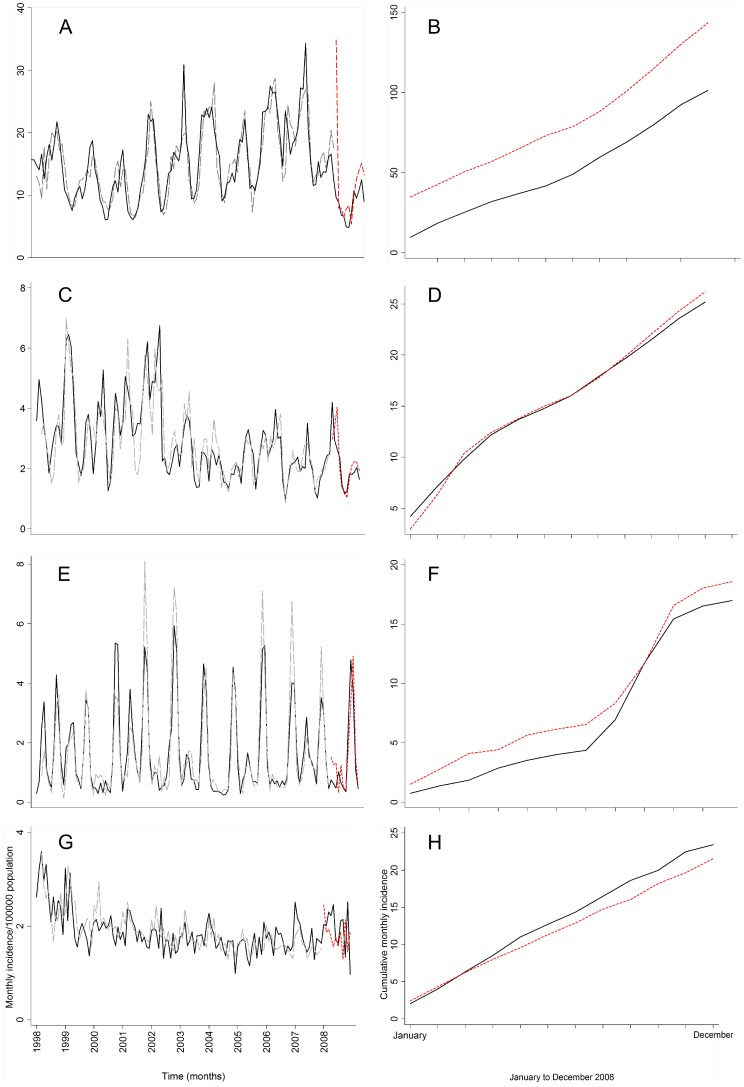
SARIMA model of forecasting weather variation in New Zealand (A-C-E-G). Actual monthly incidence /100000 population (black line), rates predicted by the chosen SARIMA models for each disease (grey dashed line) and rates predicted for the validation period ( January to December 2008) (red dashed line). (B-D-F-H) Cumulative monthly incidence /100000 population of the actual rates (black line) and rates predicted by the chosen SARIMA models for each disease (red dashed line) from January to December 2008 (validation period). Campylobacteriosis (A-B), salmonellosis (C-D), cryptosporidiosis (E-F), giardiasis (G-H). The y axis gives the monthly incidence and the x axis represents time in months.

Out-of-sample predictions for the year 2008 were compared with the observations. The chosen models (SARIMA (1, 0, 0) (2, 0, 0)_12_ for campylobacteriosis, SARIMA (1, 0, 0) (0, 0, 1)_12_ for salmonellosis, SARIMA (1, 0, 0) (1, 0, 0)_12_ for cryptosporidiosis and SARIMA (1, 0, 0) (1, 0, 1)_12_ for giardiasis) were realistically appropriate models for forecasting incidence (Figure 3). Results of the Diebold-Mariano test for forecasting accuracy indicated that for salmonellosis and cryptosporidiosis the multivariate models were better, while values for the unadjusted and multivariate models for campylobacteriosis and giardiasis were not significantly different from each other (Table 6). Finally, a plot of the predicted and actual rates along with the cumulative sums of actual and predicted values for disease incidence showed that the models were a reasonable fit (Figure 3).

**Table 6:** Forecasting accuracy of SARIMA unadjusted and multivariate (with climatic predictors) models for all four diseases.

	Unadjusted		Multivariate	
	MAPE	MAE	MAPE	MAE
Campylobacteriosis	1.29	0.16	1.28	0.16
Salmonellosis	1.01	0.21	0.90	0.19*
Cryptosporidiosis	1.17	0.31	1.14*	0.33*
Giardiasis	1.41	0.14	1.33	0.14

* Significant at the 0.05 level.

## Discussion

The findings from our study suggest that inter annual climate variability, indicated by the ENSO phenomenon (measured by the SOI) in association with regional temperature and precipitation has a general influence on enteric disease incidence. In particular, we have shown that regional climatic factors are significant predictors of salmonellosis and cryptosporidiosis but not campylobacteriosis or giardiasis.

Temperature and SOI of the current month and SOI of the previous month were positively and significantly associated with monthly salmonellosis incidence, with a dominant summer peak in cases (Figure 1b). These results are consistent with previous research, globally [18] and in New Zealand [20]. Due to the ENSO driven teleconnection patterns for New Zealand, positive SOI or *La Niña* like conditions are typically characterised by anomalous north-easterly airflows [33], bringing warmer, wetter weather to most of the country [34]. Given the thermophilic nature of *Salmonella* spp. [35], an increase in summer temperature could increase pathogen multiplication [36]. This increased pathogen load could subsequently be easily spread through food [37], water [38] or contaminated environments [39]. As food is the dominant source of *Salmonella* in many countries [40], [41], an increase in summer temperatures could increase the risk of food-borne transmission. There may be a temporal lag (delay) between climate variation and increase in disease notification where contamination is related to food production or distribution [42]. Sustained warmer temperatures could increase length of transmission seasons, enhancing opportunities for food handling errors leading to enteric disease outbreaks [43]. Indeed, enhanced food hygiene regulations over time may be partly responsible for recent weakening of the relationship of salmonellosis with temperature in New Zealand [20].

Livestock are also an important enteric pathogen reservoir in New Zealand with salmonellosis (*Salmonella* brandenburg) incidence in humans related to the lambing season [44]. Therefore, it is plausible that agricultural runoff and subsequent contamination of drinking water supplies has a role to play in disease transmission. Private well water, prone to contamination has been associated with increased salmonellosis risk [45]. Such a transmission pathway would also result in a delayed disease response. Interestingly, despite the distinct epidemiologies of different *Salmonella* serotypes [46], broad associations with regional climate were detected in our study. Nonetheless, this delayed effect is similar to those reporting positive lagged relationships between salmonellosis and temperature in England and Wales [18], [47], Canada [48] and Australia [49].

Analysis of long term data in New Zealand shows a 0.5°C warming since 1950 [50], a decrease in the diurnal temperature range [51], fewer days with temperatures below 0°C and an increase in the number of days with temperatures above 30°C in warmer locations [52]. Although sub national patterns in the intensity and range of these effects are acknowledged, based on the results of the present study, increasing temperatures in New Zealand could favour higher *Salmonella* loads in the environment due to the well-established link between bacteria and temperature. At the same time, warmer temperatures could also affect social habits such as increased outdoor activity, potentially enhancing opportunities for *Salmonella* transmission. In New Zealand, a 15% increase in salmonellosis for every 1°C rise in average monthly temperature has been predicted [20]. Similarly, in Australia, compared with the Years Lost due to Disabilities (YLDs) in 2000, “increasing temperature and demographic changes may lead to a 9%–48% increase in the YLDs for *Salmonella* infection by 2030 and a 31%–87% increase by 2050 in the temperate region, and a 51%–100% increase by 2030 and an 87%–143% increase by 2050 in the subtropical region, if other factors remain constant” [53]. Importantly, in Europe, projections of the economic costs of additional cases of salmonellosis resulting from climate change effects in the period 2071–2100 range from 140 million Euro to 280 million/year [54]. Taken together, these findings suggest the potential for salmonellosis to become a major social and economic liability as a consequence of climatic changes in New Zealand.

For cryptosporidiosis, average temperature of the previous month was positively associated with monthly incidence, while SOI two months previously was inversely related with incidence. A similar positive association with temperature of the previous month has been reported in the United States [4], Australia [25] the United Kingdom [55] and previously in New Zealand [22]. In the previous New Zealand study, authors suggested that recreational activities such as swimming and outdoor camping may be important in driving transmission in autumn (time of year when an association with temperature was reported [22]).

A spring peak in cryptosporidiosis (Figure 1c) is thought to be caused by agricultural practices, notably the birth of livestock [56]. Significantly higher cryptosporidiosis rates in rural areas [21] and outbreaks linked to farm visits [57] support this. Thus, the positive association with temperature could be due to an indirect effect of climate, whereby seasonal exposure to high pathogen loads typically takes place in warmer conditions. Conversely, the spring season is when the strongest linear relationship between the state of the Southern Oscillation (measured by the SOI) and New Zealand temperature and precipitation anomalies is seen [58]. Although the lagged response may be attributed to reporting delays, we attempted to minimise this bias by using the case onset date rather than reporting date.

The negative association of cryptosporidiosis with SOI in this study suggests a link with *El Niño* like conditions, which, in New Zealand, are typically characterised by an increased frequency of cold south-westerly airflows [34], leading to decreased temperatures and drier than usual conditions [59]. This could have important implications for the predominantly waterborne *Cryptosporidium* spp. In England and Wales, 20% of waterborne disease outbreaks in the twentieth century were due to extended periods of low rainfall, as opposed to 10% associated with heavy rainfall [61]. A global analysis of diarrhoeal incidence in children found a negative linear association with rainfall [62], consistent with the results of a cross-sectional study in the Pacific Islands [63]. In Australia, a negative relationship between weekly rainfall, relative humidity, and cryptosporidiosis incidence, and a positive association with temperature, suggest that extended dry periods may also affect transmission [64]. Negative values of the SOI are linked to reduced peak flow and flood frequency in major river systems in New Zealand [60]. Droughts or prolonged dry periods can lead to greater effluent pathogen concentration in water sources which can be flushed out by subsequent periods of rainfall. Such conditions have been known to overwhelm water supply infrastructure in the past leading to cryptosporidiosis outbreaks [7], [65]. Transmission through waterways may also partly explain the lag between SOI and disease incidence found here.

Consistent with global trends attributed to anthropogenic climate change, increased variability in rainfall patterns and drought intensity have been observed in New Zealand [52]. Moreover, relationships between river flow regimes and interdecadal climatic changes [66] as well as a relationship between SOI and water quality (independent of changes in flow linked to rainfall variability) [67] have been reported. Such changes may be influential in driving future patterns of waterborne cryptosporidiosis. However, the effect of rainfall on disease incidence can be modified by the quality of drinking water supplies, with better quality drinking water providing a protective effect [21]. Thus, improving the quality of drinking water supplies is likely to be a useful adaptation measure against expected rainfall extremes.

Changes in rainfall patterns could also have important consequences for New Zealand pasture production [68]. Given the importance of zoonotic transmission from livestock [56], changing pasture production could result in geographical shifts in agricultural systems/practices with follow on effects for health. Exploring the influence of interactions between climatic and land use processes on enteric disease risk would provide a useful baseline for future enteric disease projections.

The lack of an apparent relationship of climatic factors with campylobacteriosis is in keeping with the literature which shows mixed results. In Australia an inverse relationship between weekly temperature and campylobacteriosis cases in Adelaide was shown, while a positive relationship was reported in Brisbane [69]. Previously, a spatial analysis of campylobacteriosis determinants in New Zealand found that climate was not significantly associated with the rate of human infections [70], [71]. A detailed examination of campylobacteriosis seasonality in New Zealand and other European countries found that the seasonal peak in New Zealand was the most variable [72], indicating a seasonal trigger that may be unrelated to climate. One possible reason for this finding is that historically, retail poultry has been the dominant source of *Campylobacter* infection in humans [73], with infections being positively associated with consumption of inadequately cooked chicken [74], fast food outlet density [71] and urban residence [70]. This suggests that the summer peak seen here may be more reasonably related to activities such as summer barbequing and consumption of undercooked chicken [75], or contamination rates in chicken flock [76]. As a significant decline in campylobacteriosis in New Zealand following industry led interventions has been noted [77], it is likely that strengthening food production practices and food hygiene may be an adequate adaptation to reduce campylobacteriosis risk with climate change [78]. The drastic 54% decline in campylobacteriosis cases in 2008 compared with previous years [77] may also indicate why the model forecasts in this study did not perform that well (Figure 3).

The absence of a relationship between climate and giardiasis rates seen here is in contrast to an earlier study in which spatial patterns in giardiasis notifications were positively, albeit weakly, associated with temperature [21]. In New Zealand, high disease rates in urban areas [79] and a significant increase in infection risk linked to changing baby diapers has been reported [80]. This evidence coupled with the relatively small late summer-early autumn increase in cases (Figure 1d) suggest that human activity may be primary drivers of giardiasis incidence [16].

The topography of New Zealand plays an important role in affecting the influence of global circulation fluctuations on local temperature and rainfall patterns [34] as well as amplifying differences in regional patterns [33]. Our nationally aggregated analysis was unable to capture spatial heterogeneity in climate-disease associations [69] (Figure S1). Geographical differences in the relative importance of different transmission mechanisms were also not considered. Nonetheless, large-scale variations in atmospheric circulation in the Southern Hemisphere (represented by the SOI) influence local weather characteristics in New Zealand through their effects on rainfall extremes [81], river flow [60], temperature and mean sea level pressure [82] and annual snowlines on glaciers [83]. Thus, there is a growing body of evidence showing a link between regional weather response and atmospheric circulation. This suggests that evaluating the influence of inter-annual climate variability in association with regional climate may help identify disease sensitivity to future changes in global and local climate.

Counter-intuitively, large scale climate indices, of which the SOI is one, can outperform local climatic factors when predicting species’ dynamics [84]. While local measures of climate may fail to capture the complexity of the relationships between local weather and ecological processes, climate indices may reflect these associations better, although incompletely [84]. As climate indices represent a composite of climatic variables it may be argued that they are better indicators of natural climate variation than single weather variables [85]. In New Zealand, ENSO related variations in atmospheric circulation are dominant drivers of regional temperature and precipitation patterns [86].

This study has implications for development of adaptation strategies in response to predicted climates. Adaptation responses such as environmental and food safety regulations are more applicable at a regional scale as opposed to a local community scale. ENSO parameters may contribute to development of early warning systems for enteric diseases. Such systems would probably provide the most useful predictions during *El Niño* or *La Niña* events. The fact that *El Niño* events can often be forecast several months in advance can increase the prediction lead time for early warning systems based on ENSO parameters. Although the link between atmospheric circulation and regional temperature and precipitation is widely acknowledged, this study highlights the limited understanding of interactions between the two in driving disease patterns. Although preliminary, this study provides key considerations for regional climate change adaptation options in New Zealand.

Our study was limited by data quality, as notification data may vary in space and time due to reporting biases and other disease surveillance artefacts. We attempted to reduce this bias by considering monthly incidence and the onset date of cases. However, no major changes were made regarding the surveillance of these notifiable diseases from 1997–2008 suggesting that the completeness of reporting is likely to have remained the same over this time period. Although notified cases represent only a portion of actual incidence in the community [87], the main aim of this study was to study the temporal association between disease incidence and climate. It seems unlikely that there were substantial seasonal variations in reporting which might have introduced bias. The patterns reported here could differ by pathogen strain [88]; however strain specific information was not available. As the analyses were done nationally, spatial heterogeneity in climate could not be accounted for. A related limitation is the temporal scale of the study. While monthly data have been used for similar studies [31], weekly data might be more appropriate, particularly to establish effects of heavy rainfall. Finally, population factors influencing disease incidence like demographics, socio-economic characteristics and immunity status and pathogen level factors like reservoirs and virulence were not considered. In New Zealand, poor communities are less likely to seek medical attention for minor enteric diseases than rich communities, meaning that the completeness of notification varies by socio economic position [89]. While disease incidence is mediated by a variety of interacting factors, hygiene practices and socio-economic factors do not vary on a monthly timescale and so cannot confound the temporal associations reported here.

## Conclusions

This study complements the understanding of the relationship between climatic variables and enteric disease incidence by characterising the association between regional weather, a measure of inter-annual climate variability and the incidence of specific enteric diseases in New Zealand. Salmonellosis was positively associated with warmer, wetter conditions while cryptosporidiosis incidence was linked to cooler, drier conditions. These results highlight regional climate forcing as a factor influencing enteric disease incidence, emphasising the potential effect of future regional climate change on enteric disease risk. Such an analysis offers insights into potential adaptation options for climate change related health impacts in New Zealand. By analysing historical disease patterns, such investigations can enhance disease prediction models [90], identify diseases that are potentially useful markers of changes in global climate or local weather [91] and contribute to the development of climate based, early warning systems. 

## Supporting Information

Figure S1 **Mean monthly temperature (B) and rainfall (C) across four cities in New Zealand (A).** The graphs show average monthly temperature and rainfall values in Nelson, Taupo and Christchurch as correlated with average monthly values in Auckland. Values in red are those for Auckland.
(TIF)
