# Supplementary figures and images for: Climate Variability, Weather and Enteric Disease Incidence in New Zealand: Time Series Analysis
Source: PLoS One. 2013 Dec 23;8(12):e83484. doi: 10.1371/journal.pone.0083484 (PMC3871872; doi:10.1371/journal.pone.0083484)

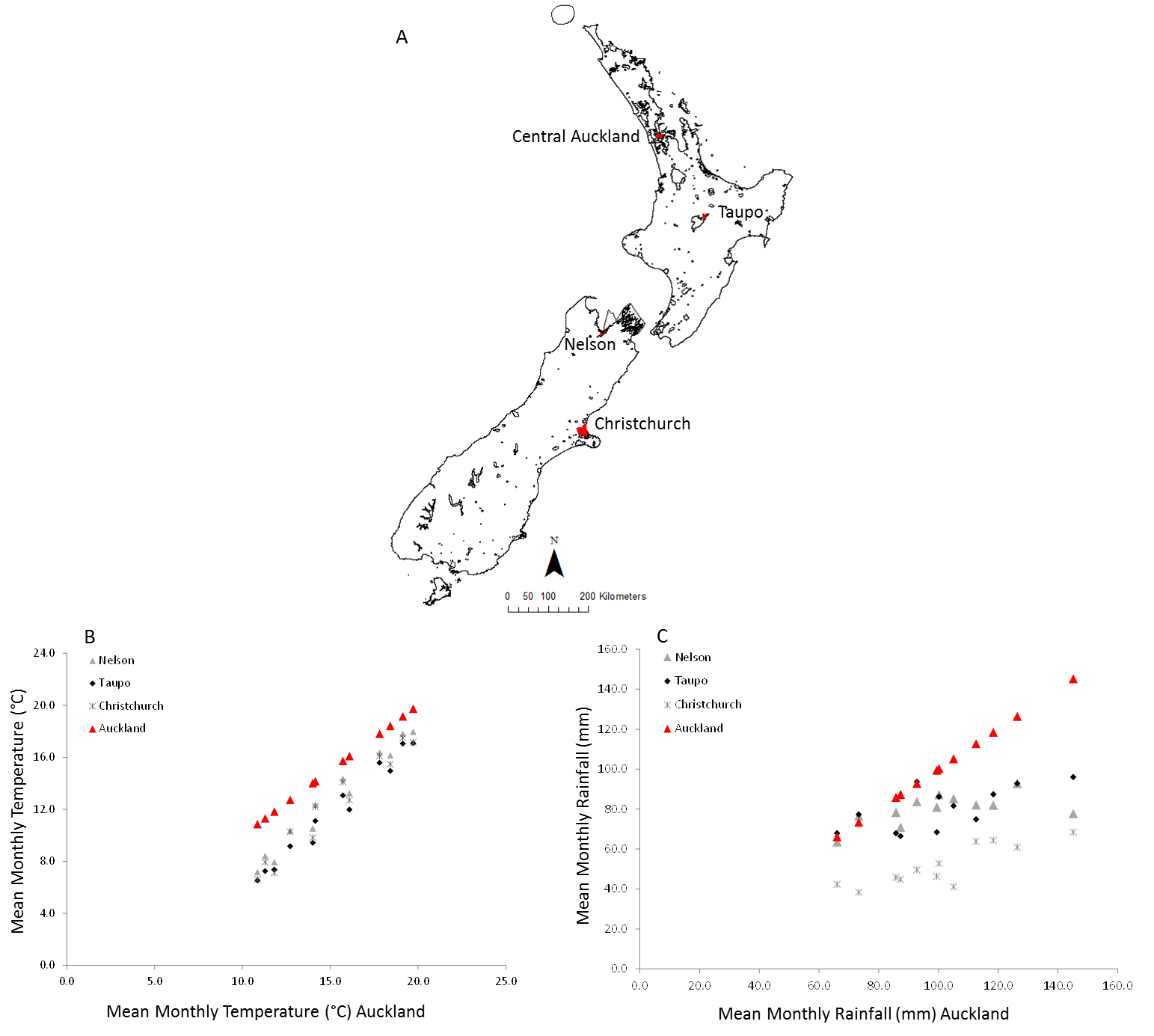

Supplement: Figure S1 — Mean monthly temperature (B) and rainfall (C) across four cities in New Zealand (A). The graphs show average monthly temperature and rainfall values in Nelson, Taupo and Christchurch as correlated with average monthly values in Auckland. Values in red are those for Auckland. (TIF) [file pone.0083484.s001.tif]
